# Toward Global Comparability of Sexual Orientation Data in Official Statistics: A Conceptual Framework of Sexual Orientation for Health Data Collection in New Zealand's Official Statistics System

**DOI:** 10.1155/2013/473451

**Published:** 2013-06-12

**Authors:** Frank Pega, Alistair Gray, Jaimie F. Veale, Diane Binson, Randell L. Sell

**Affiliations:** ^1^Department of Public Health, University of Otago, P.O. Box 7343, Wellington, New Zealand; ^2^Department of Social and Behavioral Sciences, Harvard School of Public Health, Harvard University, 677 Huntington Avenue, Boston, MA 02115, USA; ^3^Statistics Research Associates Ltd., 8 Bristol Street, Island Bay, Wellington 6023, New Zealand; ^4^Department of Psychology, University of British Columbia, 2569-2136 West Mall, Vancouver, BC, Canada V6T 1Z4; ^5^Center for AIDS Prevention Studies (CAPS), Department of Medicine, University of California San Francisco, 50 Beale Street, Suite 1300, San Francisco, CA 94105, USA; ^6^Department of Community Health and Prevention, Drexel University School of Public Health, The Bellet Building, 1505 Race Street, Philadelphia, PA 19102, USA

## Abstract

*Objective.* Effectively addressing health disparities experienced by sexual minority populations requires high-quality official data on sexual orientation. We developed a conceptual framework of sexual orientation to improve the quality of sexual orientation data in New Zealand's Official Statistics System. *Methods.* We reviewed conceptual and methodological literature, culminating in a draft framework. To improve the framework, we held focus groups and key-informant interviews with sexual minority stakeholders and producers and consumers of official statistics. An advisory board of experts provided additional guidance. *Results.* The framework proposes working definitions of the sexual orientation topic and measurement concepts, describes dimensions of the measurement concepts, discusses variables framing the measurement concepts, and outlines conceptual grey areas. *Conclusion.* The framework proposes standard definitions and concepts for the collection of official sexual orientation data in New Zealand. It presents a model for producers of official statistics in other countries, who wish to improve the quality of health data on their citizens.

## 1. Introduction

Robust evidence demonstrates that sexual minority populations^1^ have poorer access to care, poorer care, and higher morbidity than heterosexual populations in areas such as mental health, sexual health, and cancer [1–3]. Over the last decade, public health authorities and international health organisations have developed policies to eliminate these health disparities [4–8]. Effectively developing such policies and monitoring their outcomes require official data on sexual orientation that is timely, accurate, reliable, comparable, and of overall high quality [9, 10].

Producers of official health statistics throughout the world have started collecting sexual orientation data by adding sexual orientation questions in official health surveys. The United States Department of Health and Human Services has obtained data on sexual orientation in several surveys since the early 1990s [11]. The United Kingdom Office for National Statistics conducted the Sexual Identity Project from 2006 to 2009 to develop and trial sexual identity questions [12] and recently began the standard implementation of these questions in several surveys [13]. New Zealand's Official Statistics System (OSS), which comprises around 70 government departments that produce official statistics, has also gained significant experience in this area. More specifically, the Ministry of Health has conducted various health surveys which included sexual orientation questions, and Statistics New Zealand has led the initiative of considering sexual orientation as an official social (including health domain) statistic [14].

Despite these advancements in collecting official sexual orientation data, little progress has been made in developing standard definitions and concepts for these data. The United Kingdom Office for National Statistics came closest to developing such standards when it explored the concept of sexual identity (one dimension of sexual orientation) in focus groups [15]. However, to our knowledge no producer of official statistics has developed explicit standard definitions and concepts to guide the collection of survey data on sexual orientation. This is despite experts calling for the development, testing, and selection of unambiguous standard definitions and concepts as the foundation for the measurement of sexual orientation in official surveys [9, 10].

Conceptual frameworks are well-recognised tools that producers of official statistics commonly use to support measurement, data analysis, and analytical commentary. The Australian Bureau of Statistics noted that “A primary function of a framework is to “map” the conceptual terrain surrounding an area of interest […]. Frameworks can define the scope of inquiry, delineate the important concepts associated with a topic, and organize these into a logical structure” (page 15) [16]. According to the Bureau, the nature and scope of the topic, the purpose of the framework, and the perspectives of its designers ultimately determine the final content and form a framework takes. Successful frameworks are logical in structure, comprehensive but concise, dynamic and flexible to allow for change, and cognisant of other frameworks, classifications, and standards. These frameworks should also be widely accepted to promote “standards, consistency and comparability across data collections and between jurisdictions (e.g., states and countries) and sectors (e.g., public and private)” (page 15) [16].

We conducted the Sexual Orientation Data Collection Study in New Zealand on behalf of the Ministry of Social Development and its partners, the Ministry of Health and Statistics New Zealand [17]. Through this study we sought to develop a conceptual framework for the collection of sexual orientation data in official probability surveys in New Zealand that would be coherent and theoretically robust and that would adhere to OSS requirements^2^. This paper overviews the development and content of this sexual orientation conceptual framework. It then discusses the implications of the framework in the context of the emerging global movement of collecting sexual orientation data in official surveys.

## 2. Materials and Methods

We used a multimethod, multisource research approach to develop the conceptual framework of sexual orientation.

### 2.1. Literature Review and Analysis

A systematic search of academic and grey literature was carried out across various scientific databases (Academic Search Complete; International Bibliography of the Social Sciences; Ovid MEDLINE(R) 1946 to Present with Daily Update; PsycINFO; Sociological Abstracts), using a range of keywords^3^ to identify literature on the definition, conceptualisation, and measurement of sexual orientation and on the collection, analysis, and reporting of sexual orientation data in probability surveys. These searches identified 109 papers. We reviewed this literature and conducted a secondary analysis that identified key definitions and concepts of sexual orientation. We drafted a first version of the sexual orientation conceptual framework.

### 2.2. Focus Groups and Key-Informant Interviews

To ensure that the framework's development was informed by community expertise, we sought feedback on our draft from sexual minority individuals. We held 90-minute focus groups in New Zealand's two largest cities Auckland and Wellington (four participants and five participants, resp.) and one 60-minute key-informant interview with a total of ten sexual minority individuals, including takatāpui ^4^, fa'afafine ^5^, lesbians, gays, and bisexuals. Purposive sampling of the participants ensured a diversity of knowledge and opinions, with an emphasis on significant representation of indigenous (Māori) perspectives. To ensure the appropriate consideration of OSS requirements, we also conducted 60-minute key-informant interviews with four producers and consumers of official statistics. Key informants included senior policy staff from the Ministry of Health, the Ministry of Social Development, and Statistics New Zealand. We conducted a thematic analysis of the focus-group and interview data [18] to identify the key sexual orientation concepts identified by the sexual minority key informants. We used the informants' recommendations to improve the draft framework.

### 2.3. Expert Advice

The advisory board of the Sexual Orientation Data Collection Study comprised 14 national and international experts on official statistics and on the collection of sexual orientation data. This board provided us with ongoing peer review throughout the development of the sexual orientation conceptual framework. Several staff from the Ministry of Social Development and Statistics New Zealand, as well as Māori experts, including from Te Puni Kōkiri/The Ministry of Māori Development, commented on draft versions of the framework. We finalised the framework by integrating the feedback from these diverse experts.

## 3. Results and Discussion

### 3.1. Results

The structure and content of the framework are as follows. The framework

- proposes working definitions for the sexual orientation statistical topic and its measurement concepts,
- describes key dimensions of the sexual orientation measurement concepts,
- discusses important variables framing the sexual orientation measurement concepts,
- outlines conceptual grey areas.

#### 3.1.1. Scope of the Framework

The conceptual framework is about sexual orientation and hence does not discuss gender identity concepts per se. Gender identity is a conceptually distinct variable that must not be confused with sexual orientation. However, gender identity does inform and frame sexual orientation in some sexual minority groups and is therefore discussed as a variable that frames sexual orientation in the framework.

The conceptual framework is currently limited in scope to adult sexual orientation though it includes some literature on adolescent sexual orientation, where this literature is relevant to adult populations. To fully cover adolescent sexual orientation concepts, the framework may need to be extended.

#### 3.1.2. Working Definitions for the Sexual Orientation Statistical Topic and Its Measurement Concepts

Through our study we came to understand sexual orientation in the context of official statistics as a statistical topic with three key measurement concepts: sexual attraction, sexual behaviour, and sexual identity.

We reviewed existing definitions of sexual orientation but did not consider these fit for adoption by the OSS. Consequently we developed a working definition for the sexual orientation statistical topic (Table 1), which defines the key measurement concepts, their relationships, and conceptual features.

We reviewed existing definitions of the three sexual orientation measurement concepts and adopted or adapted those that promoted standards suitable for the OSS (Table 2). These definitions have previously been used in health research.

If applied consistently, the proposed definitions will contribute to the conceptual standardisation of health data on sexual orientation and thus improve the conceptual quality of these data. These working definitions should be developed further as scientific knowledge and experience in this area advance.

#### 3.1.3. Key Dimensions of Sexual Orientation Measurement Concepts

*(1) The Measurement Concepts.* The working definitions differentiate three key dimensions of sexual orientation: sexual attraction, sexual behaviour, and sexual identity. The academic literature generally suggests that sexual orientation comprises these concepts [19, 21–23]. Some definitions of sexual orientation include other concepts, including sexual fantasy and sexual desire. However, such concepts overlap the three measurement concepts proposed in the framework or are peripheral to the conceptualisation of sexual orientation for official data collection purposes.

*(2) The Relationship between the Measurement Concepts.* The three concepts overlap considerably, but substantial research demonstrates that individuals often do not report consistently across them. For example, many women who report sexual attraction to women engage in same-sex behaviour, but others do not [24]. Sexual attraction can, but does not necessarily, result in various sexual behaviour and the adoption of sexual identities [25]. Multiple survey questions need to be asked to differentiate the populations encompassed by the three key measurement concepts.

*(3) The Continuous Nature of Sexual Attraction and the Categorical Nature of Sexual Behaviour and Identity.* Sexual attraction to men and women can be conceptualised along two separate continuous scales marked by the extreme poles of “not at all sexually attracted” and “extremely sexually attracted” [26]. Asexuality (defined as the absence of sexual attraction [27]) is indicated by low scores on both scales. Official questions that measure sexual attraction along a continuum acknowledge that sexual attraction is a continuous variable [19, 28, 29]. Data from such questions is likely to be more accurate than data from questions using distinct categories.

Sexual behaviour and identity are categorical variables. For official statistical purposes of measuring sexual orientation, sexual behaviour is best understood as having four categories (i.e., opposite-sex behaved, both-sex behaved, same-sex behaved, and not sexually active). Sexual identity categories are descriptive and nominal.

*(4) The Fluidity of Sexual Attraction*, *Behaviour, and Identity.* The sexual attraction, behaviour, and/or identity of some individuals change over time and across social contexts. For example, one longitudinal study found that sexual attraction was unstable among young adults, who experienced some same-sex sexual attraction, especially amongst women [30]. Those with major same-sex attraction experienced less change in sexual attraction over time [30]. 

Several phenomena and processes explain and underlie the fluidity of sexual orientation. Two crucial factors that can lead to changes in sexual attraction, behaviour, and identity are an individual's level of certainty about their sexual attraction and identity and differences in the extent of their exploration of sexual attraction, behaviour, and identity [19, 31, 32]. Furthermore, some individuals choose not to label their sexual identity [33]. Others adopt alternative sociopolitical sexual identities, such as *queer*, which challenge more traditional, rigid ones [34]. The emergence of sexual identity categories such as those that incorporate interrelated gender, gender-identity, and gender-role dimensions also indicates that sexual identity terminology changes and has become increasingly complex over time [31, 33, 35].

*(5) Multiple Current Sexual Identities.* An important issue for the measurement of sexual identity is the adoption of multiple current sexual identities (i.e., more than one identity at the same time). Māori [36] and Pacific sexual minority people [37] commonly adopt and manage multiple sexual identities. To address this issue, the OSS can draw on its vast experience in measuring other multiple current social identities, such as multiple ethnic identities [38].

#### 3.1.4. Variables Framing Sexual Orientation Measurement Concepts

The framework considers sexual orientation in the context of cultural and gender-related variables.

*(1) Cultural Variables.* In terms of culture, sizeable ethnic groups in New Zealand differ in their understanding of sexual orientation. New Zealand Māori are familiar with sexual orientation as a concept and with its three measurement concepts. They tend to report Māori-specific minority sexual identities used historically and in contemporary times (i.e., *takatāpui wāhine* ^6^ and *takatāpui tāne* ^7^), as well as common Western sexual identities (e.g., *straight/heterosexual, gay, lesbian,* and *bisexual)*, sometimes alongside each other [36, 39–45]. New Zealand Europeans/Pākehā are also familiar with sexual orientation as a concept and its three measurement concepts. They frequently adopt common Western sexual identity labels [36]. People from Pacific Island backgrounds traditionally do not appear to be familiar with the concepts of sexual orientation and sexual identity. However, same-sex sexual attraction and behaviour are relatively common among young, unmarried Pacific men. Moreover, some Pacific people who engage in male same-sex sexual relationships adopt specific sexual or social identities (e.g., Samoan *fa'afafine*) [37, 46]. Some people from Asian backgrounds do not have traditional cultural concepts equivalent to Western sexual orientation and sexual identity. However, same-sex attraction and behaviour are not uncommon amongst these groups in New Zealand [47]. New migrants are inclined to adopt the sexual orientation concepts of the host culture. Whether they do or not depends on several factors, including country of origin and level of acculturation to the host culture [33, 48, 49]. These examples illustrate that cultural understandings of sexual orientation vary in New Zealand. The understandings of sizeable populations need to be considered when official standard measures of sexual orientation are developed.

The framework could potentially be extended to include cultural factors other than ethnicity such as national customs, religion, and globalisation. As more details will be added to the framework over time, a discussion of which specific measurement concept is shaped by which specific cultural variable might be valuable. In any case, the OSS requires that official statistics span across cultures. Therefore, the conceptual framework would benefit from additional research that could determine whether sexual orientation is a concept well understood by all sizeable New Zealand populations.

*(2) Gender-Related Variables.* Gender-related factors also frame the understanding of sexual orientation. Differences in how men and women experience their sexual orientation have been described by Golden (see [22, 50]) and others (e.g., [51]). Whether Pacific males adopt a gender-typical or gender-atypical role can influence how they conceptualise their sexual attraction, behaviour, and identity [37]. Similarly, whether an individual takes an insertive (active) or receptive (passive) role during (same-sex) sexual behaviour can affect how they conceptualise their sexual identity [37, 49, 50]. For example, taking a receptive role during sexual activity is associated with the adoption of a sexual minority identity (such as *fa'afafine* among Samoan males), whereas taking an insertive role is not [37].

Finally, gender identity is an important consideration when conceptualising sexual orientation. Some transgender people conceptualise their sexual attraction, behaviour, and identity differently depending on whether they predicate these on their (biological) sex or gender identity [15, 37, 52, 53]. If a sexual orientation question does not specify whether it expects respondents to base their response on biological sex or gender identity, then transgender people are likely to respond to it in relation to their gender identity [54]. However, in one official health survey conducted in the United States, transgender participants reported their sexual orientation in relation to their biological sex because they assumed that biological sex was more important than gender identity in a health context (Sell, unpublished). This highlights that some transgender people may consider the survey context when deciding which variable to base their sexual orientation on, while others explicitly reject the notion that they must necessarily base their responses on an assessment of the potential relevance of their assigned sex. Also, some do not identify completely with traditional gender identities of “male” and “female.” For these people and for those who are discovering or coming to terms with their gender identity, traditional sexual orientation identities that assume a stable male or female gender identity (such as *heterosexual/straight*, *gay,* and l*esbian*) are likely to be inappropriate.

#### 3.1.5. Conceptual Grey Areas

The framework highlights the following culture-specific conceptualisations of sexual orientation that require further investigation:

- Māori preferences relating to sexual identity categories.
- Pacific people's understandings of the sexual orientation topic and of their sexual identity concepts, including especially the Samoan fa'afafine concept ^8^.
- Asian people's understandings of the sexual orientation topic and of their sexual identity concepts.

To summarise, the conceptual framework does not conceptualise gender identity. Rather, it identifies gender identity as a variable that frames sexual orientation. Additional research on this variable would be helpful. Topics of special interest include comparing:

- how transgender and non-transgender people understand their sexual attraction, behaviour, and identity
- how survey respondents conceptualise sexual attraction to and behaviour with transgender and non-transgender people.

While this research would be conceptually relevant, we acknowledge the presumed very small estimated size of the transgender population in New Zealand [55].

### 3.2. Discussion

Nationally, producers of official health statistics currently engage in or are in the process of setting up the routine collection of sexual orientation data as the basis for robust health policy making and outcomes monitoring. Broadly applicable, nationally (and potentially internationally) agreed-upon standard definitions and concepts for sexual orientation need to be established to ensure the quality and comparability of the data obtained. 

This paper has described the development and content of a conceptual framework of sexual orientation tailored to the New Zealand context and OSS requirements. The United Kingdom Office for National Statistics has gone some way towards establishing a standard definition and measurement concept for sexual identity [15]. However, to our knowledge the conceptual framework described in this paper is the first statistical framework that establishes standard definitions and concepts for the broader sexual orientation statistical topic, which can be applied in an official statistics system.

The framework synthesises a large body of contemporary research on the definition, conceptualisation, and measurement of sexual orientation and on the collection, analysis, and reporting of sexual orientation data in probability surveys. The framework has been developed by a small team of researchers but has been peer-reviewed rigorously throughout its development. It has not undergone formal public consultation to date, but its development has been guided by feedback from a small number of demographically diverse individuals with sexual minority orientations, Māori reviewers, and a considerable number of producers and consumers of official statistics. The advisory board of the Sexual Orientation Data Collection Study also gave feedback, which has been integrated.

The validity of the framework and the standards it sets will only be established over time, as the framework is applied in the OSS. One initial milestone in applying the framework will be the development of standard questions on sexual attraction, behaviour, and identity for use in official surveys. The framework could also form the foundation for developing standard statistical classifications for the three sexual orientation measurement concepts. Additionally, it could be used to guide data commentary, including the evaluation of the quality of sexual orientation data. These next steps will validate the framework and identify areas that require improvements.

The scope of the current study was to define, conceptualise, and measure a specific statistical concept (i.e., sexual orientation), for New Zealand's OSS. This study identified how cultural and gender-related variables frame sexual orientation and considered how sexual identity fluidity and multiple identities might influence responding to OSS sexual orientation questions. Gender identity was a consideration in all of these discussions. For example, *queer* and *fa'afafine* identities incorporate gender role and gender identity components. There is a growing understanding of a complex interaction between gender identity and sexual orientation [56]. Given this, there is a need for future research to investigate collecting gender identity data for official statistics alongside sexual orientation data. Such research could build on the findings of the present research and others (e.g., [15]).

Globally, as more producers of official statistics collect data on sexual orientation, the cross-country comparability of these data will need to be assured through international coordination and standard setting. We believe that the standard definitions and concepts proposed in our framework are potentially transferable to some countries, especially those that are historically and demographically similar to New Zealand such as Australia and the United States. These countries also have sizeable Indigenous and Pacific Island populations that require consideration of concepts and categories other than the common Western ones. 

Other countries will need to develop equivalent frameworks of sexual orientation to suit their specific contexts and the requirements of their producers and consumers of official statistics. The producers of official statistics will need to establish a systematic process that allows them to share information related to the definition and conceptualisation of sexual orientation. This will enable them to compare across countries the sexual orientation data that they collect.

## 4. Conclusions

We have developed a coherent and theoretically robust conceptual framework that sets standard definitions and concepts for collecting official sexual orientation data in New Zealand's OSS. The validity of the framework and the standards it sets will be established over time, as the framework is applied in the OSS. The framework is potentially transferable to official statistics systems in countries historically and demographically similar to New Zealand. The framework offers a first model that could help improve the quality of sexual orientation data collected in official surveys.

## Figures and Tables

**Table 1:** Proposed working definitions for the sexual orientation statistical topic.

Statistical topic	Proposed working definition
Sexual orientation	Sexual orientation encompasses three key concepts: sexual attraction, sexual behaviour, and sexual identity. The relationship between these components is that sexual orientation is generally based upon sexual attraction and that sexual attraction can result in various sexual behaviour and the adoption of sexual identities. The three key concepts are related, but not necessarily congruent variables, each of which can independently change over time and by social context.

**Table 2:** Proposed working definitions for the sexual orientation measurement concepts.

Measurement concept	Proposed working definition	Source
Sexual attraction	The desire to have sexual relationships or to be in a primary loving, sexual relationship with one or both sexes.	Savin-Williams (page 41) [19]

Sexual behaviour	Any mutually voluntary activity […] that involves genital contact (for at least one of the partners) and sexual excitement or arousal, that is feeling really turned on, even if intercourse or orgasm did not occur.	Laumann et al. (page 67) [20], text in brackets added by authors

Sexual identity	Personally selected, socially, and historically bound labels attached to the perceptions and meanings individuals have about their sexuality	Savin-Williams (page 41) [19]

## Endnotes

1. Population groups defined by a minority sexual attraction, minority sexual behaviour, and/or minority sexual identity.

2. OSS social statistics are required to have a high degree of relevance by meeting user needs and to be able to inform decision making in relation to improving New Zealand's social wellbeing. All official social statistics must “address issues of enduring concern to government departments, local authorities, businesses, and to the general public; be useful for improving knowledge about New Zealand's population; inform decision making relating to New Zealand's social wellbeing; provide an accurate reflection of the population of interest; provide information that will help inform and evaluate policy; be a trusted source; be publicly acceptable; be accurate and of high quality” (page 5-6) [14]. Furthermore, the OSS is also required to consider the temporal and psychological burden that the inclusion of sexual orientation questions in official surveys will put on survey respondents; to collect concepts which span cultural understanding; and, given financial constraints, to balance the need for the collection of sexual orientation data against the need for collection of data on other topics.

3. Any combination of (1) sexual orientation, sexual identity, sexual behaviour, sexual attraction, lesbian, gay, bisexual, homosexual, homosexuality, heterosexual, heterosexuality, fa'fafine, fakaleiti, takataapui, and two-spirit and (2) sampling, sample, probability, opportunity, random, data collection, measurement, data source, census, representative, general population, population based, epidemiological, demographic, geographic, geography, distribution, prevalence, morbidity, mortality, data analysis, statistical model, base line data, and validity.

4. The indigenous, Māori language term “takatāpui” in its historical usage described “an intimate companion of the same sex”; today, the term “embraces all non-heterosexual forms of [Māori] sexuality” (words in brackets added by author; page 1 [39]).

5.
*Fa'afafine* is a Samoan language term describing the third gender identity specific to Samoa. The term is commonly translated as “in the manner of a woman.” In New Zealand, the term is sometimes used by people from Samoan backgrounds to describe a minority sexual identity [37, 57].

6.
*Takatāpui wāhine* is an identity adopted by some Māori women with a sexual orientation other than heterosexual.

7.
*Takatāpui tāne* is an identity adopted by some Māori men with a sexual orientation other than heterosexual.

8. Emphasis is placed on the Samoan ethnic group because this group constitutes a sizeable Pacific population in New Zealand.
